# Protein-Protein Interaction Analysis of Common Top Genes in Obsessive-Compulsive Disorder (OCD) and Schizophrenia: Towards New Drug Approach Obsessive-Compulsive disorder (OCD) and Schizophrenia Comorbidity Gene Analysis

**Published:** 2018

**Authors:** Mohammad Mahboubi, Mona Zamanian Azodi, Mostafa Rezaei Tavirani, Vahid Mansouri, Nayeb Ali Ahmadi, Mostafa Hamdieh, Majid Rezaei Tavirani, Bahar Naghavi Gargari

**Affiliations:** a *Abadan School of Medical Sciences, Abadan, Iran. *; b *Proteomics Research Center, Faculty of Paramedical Sciences, Shahid Beheshti University of Medical Sciences, Tehran, Iran.*; c *Psychosomatic Department, Taleghani Hospital, Faculty of Medicine, Shahid Beheshti University of Medical Sciences, Tehran, Iran. *; d *Surgery department, Faculty of Medicine, Iran University of Medical Sciences, Tehran, Iran.*; e *Basic Science Department, Shahid Beheshti University of Medical Sciences, Tehran, Iran.*

**Keywords:** Obsessive-Compulsive Disorder (OCD), Schizophrenia, Protein-Protein Interaction (1) Network, Clustering methods, ESR1

## Abstract

Comorbidty is common among psychiatric disorders including obsessive-compulsive disorder and schizophrenia with a high rate. Many studies suggested that the disorders may have same etiological bases. In this regard, shared pathways of glutamate, dopaminergic, and serotonin are the known ones. Here, the common significant genes are examined to understand the possible molecular origin of the disorders in terms of sequence and functional features. Exploring the underling mechanisms of OCD and schizophrenia is important to achieve a better treatment options. Methods of Cytoscape software following R statistical software were applied for this purpose. Needleman-Wunsch global alignment algorithm was used to determine pair-wise similarities followed by clustering methods, AGNES and PAM in R statistical programming software. The results indicate that SLC1A1, DRD2, DRD4, BDNF, ESR1, CDH2, GRIN2B, TNFa, GABBR1, and OLIG2 are significantly common for the two disorders and PPI network analysis showed the important key genes in the interaction profile. ESR1 (estrogen receptor α) as a key hub-bottleneck gene regulates many underling mechanisms of the brain. Application of global alignments indicates some of the genes with sequence similarities also elucidate similar biological terms.

## Introduction

Psychiatric disorders affect people around the world noticeably. Some of them tend to manifest together known as comorbidity conditions (2). OCD and schizophrenia as widespread mental disorders are one of the important co-occurrence types (3). OCD is identified by intrusive thoughts and the urge for reducing the unpleasant experience by different types of compulsions (4). The patients suffer from recurrent invasive thoughts or stereotype behaviors at least one hour per day (5). It is with the prevalence of about 1-3% in human population (6, 7). On the other hand, schizophrenia is a sever condition of consciousness abnormalities including hallucinations, delusions, disorganized communication, poor planning, reduced motivation, and blunted affect with 0.5% and 1% lifetime worldwide prevalence (8, 9). Both disorders have demonstrated heterogeneity in genetic concept and clinical phenotype (10, 11). About 30.3% of patients diagnosed with schizophrenia have the obsessive-compulsive symptoms (OCS) and about 13.6% of them shows co-occurrence with OCD (12).  In fact, OCD can be accounted as schizophrenia risk factor (3). There are some common features between OCD and schizophrenia despite the different diagnostic entities. Some symptoms, age of onset, gender, and structural impairment in brain including areas of prefrontal cortex, anterior cingulate, caudate nucleus and thalamus are shared in OCD and schizophrenia. The patients with two disorders, schizo-OCD, have more difficulties in prognosis, treatment responses, and lower quality of life (13). In addition, some of the applied therapeutic methods are also similar (14). Therefore, the disorders may have same molecular origin as they have a tendency of comorbidity (15). Some pathways of serotonin, dopamine and glutamine are reported common between two disorders. The most applied medications for schizophrenia-OCD comorbidity belong to selective serotonin re-uptake inhibitors (SSRI) family (16, 17). It must be noted that some of patients were not responded to treatment. In addition, some other types of drugs affecting on dopamine receptors (antagonism) also showed effective in patients with OCD (18). Antipsychotic medications, which substantially act as dopamine antagonism, are also effective in patients with schizophrenia (19). Particularly, Clozapine and Olanzapine as dopamine antagonism seemed valuable with or without SSRIs application in some schizophrenia patients (20). Both drugs act as D2 receptors antagonisms in the mesolimbic pathway; in addition, they have high affinity to 5-HT2A receptors in the frontal cortex as well (21). However, Clozapine and Olanzapine can increase the risk of obsessive-compulsive symptoms in schizophrenia patients (22). Proteomics, genomics, and protein-protein interaction (1) network analysis can provide a new insight of interactome profile of the two disorders (23, 24). Central genes in OCD comorbid schizophrenia PPI network are essentials in the network integrity and can be targets for more analysis (25). Since there are crucial genes among query and added genes which play significant role in disorder PPI network analysis is a useful tool to explore the critical genes. It is essential to evaluate the structural and functional properties of the related significant genes to decipher the core underling mechanisms of pathogenesis of the OCD and schizophrenia. Furthermore, the analysis of essential genes in the network can provide a new insight in drug targeting strategies. 

## Experimental

The associated genes with development of OCD and schizophrenia were searched from Google Scholar and PubMed. There are many reported candidate genes for the two types of disorders; however, only limited numbers were significantly common between two disorders. In fact, based on previous investigation, about 39 genes are susceptible to pathogenicity of OCD which among them only 24 candidates show significant relationship with the disorder. The individually emphasized genes for OCD and schizophrenia in the literature were selected and used for more evaluation. About ten polymorphism genes showed significant associations. The Uniprot accession codes were downloaded from http://www.uniprot.org/. Further analysis including PPI network study and sequence global comparison was handled by the applied codes. PPI network was constructed by the use of PSICQUIC (Proteomics Standard Initiative Common QUery InterfaCe) in Cytoscape v: 3.3.0., from public database including Mentha, Reactome-Fls, and Reactome. This algorithm is a universal web service client for importing interactions from public databases (26). Network Analyzer provides topological features including two used in this study, degree and betweenness centralities. This plug-in is integrated into Cytoscape and can provide different types of centrality properties of the analyzed network (27). In the PPI network, the nodes represent genes, while the edges represent physical interactions (28). The number of connected edges to the nodes indicates the degree value of that element. In addition, another evaluated centrality in the network, betweenness centrality signifies the number of shortest path that pass through each node (27). Genes with high betweenness centrality are known as bottlenecks, which can be as essential as hubs. Bottlenecks can connect many protein complexes and processes (29). For gene ontology examination, ClueGO+CluePedia plug-ins in Cytoscape can be helpful. The application explored the biological terms of the investigated genes (30, 31). GO is the reference of controlled vocabulary of the terms of three biological annotations including cellular component, molecular function, and biological process (1). There are some available related statistical features in ClueGo+CluePedia plug-ins. Kappa score was the statistical method for this analysis. The higher the score, the better the terms are grouped significantly together. Kappa score was set to 0.5. Furthermore, other setting was as follows: number of genes per term 2, percent of related genes in each term, 1 and 2 and the minimum and maximum level of ontology was set to 3 to 8 as default options, respectively. The *P*-value was also set to ≤ 0.05. The correction method for *p*-value was Bonferroni step down method and the enrichment/depletion test for terms was set to Two-sided (enrichment/depletion) based on hypergeometric. The assessment of sequence similarity of the examined genes with R programming software was another part of the conducted research. By the use of R, clustering profile of the ten genes can be understood. R software is free statistical software available on (www.r-project.org). It possess various packages, one of them is the package cluster can be used to identify subgraphs with maximal density (32). Protein sequences were extracted from Uniprot in FASTA format and then the global pairwise alignment was performed using Needleman-Wunsch algorithm, available in EMBOSS Needle, EBI Database. The construction of similarity matrix by the alignment similarity scores is provided by the alignment. The scores were then deducted from 1, as the dissimilarity matrix is need for clustering purposes in R studio software (Dissimilarity =1-similarity) (33). Agglomerative hierarchical clustering analysis (bottom up) as hierarchical and partitioning around medoids (PAM) non-hierarchical clustering algorithms were the conducted clustering methods. The distance between each cluster is calculated as D = 1-C, where D = distance and C = correlation between spot clusters. Average linkage (UPGMA) is the used method to define the distance between clusters as the average distance**. **Moreover, agglomerative coefficient signifies clustering structures strength and quality of the dataset. The calculating range is 0 to 1. The agglomerative coefficient (AC) close to 1, shows on good structured cluster, whereas AC close to 0 implies on not a powerful clustering structure (34). PAM clustering finds K representative objects called “medoids” from the dataset. In other words, data structures are represented by medoids as the representatives of the clusters (35). AGNES and PAM were the functions of clustering algorithms in this study. Dendrogram and silhouette plots are the graphical representations of clustering in this study for AGNES and PAM clustering algorithms, respectively. Peter J. Rousseeuw in 1986 presented a new graphical display called “silhouette”. This type of plot shows that which elements belong to their cluster strongly and which are in between clusters. The average silhouette width is obtained by mean of each cluster silhouette width value, which is from 0 to1 and implies on the clustering structure strength. In addition, it is useful for selecting the K (numbers of clusters) in silhouette (36). Validation of clustering algorithms was handled by different indices. 

## Results

Genes with significant correlation with OCD and schizophrenia are gathered from literature resources (Google Scholar and PubMed) (see Table 1).

**Figure 1:**
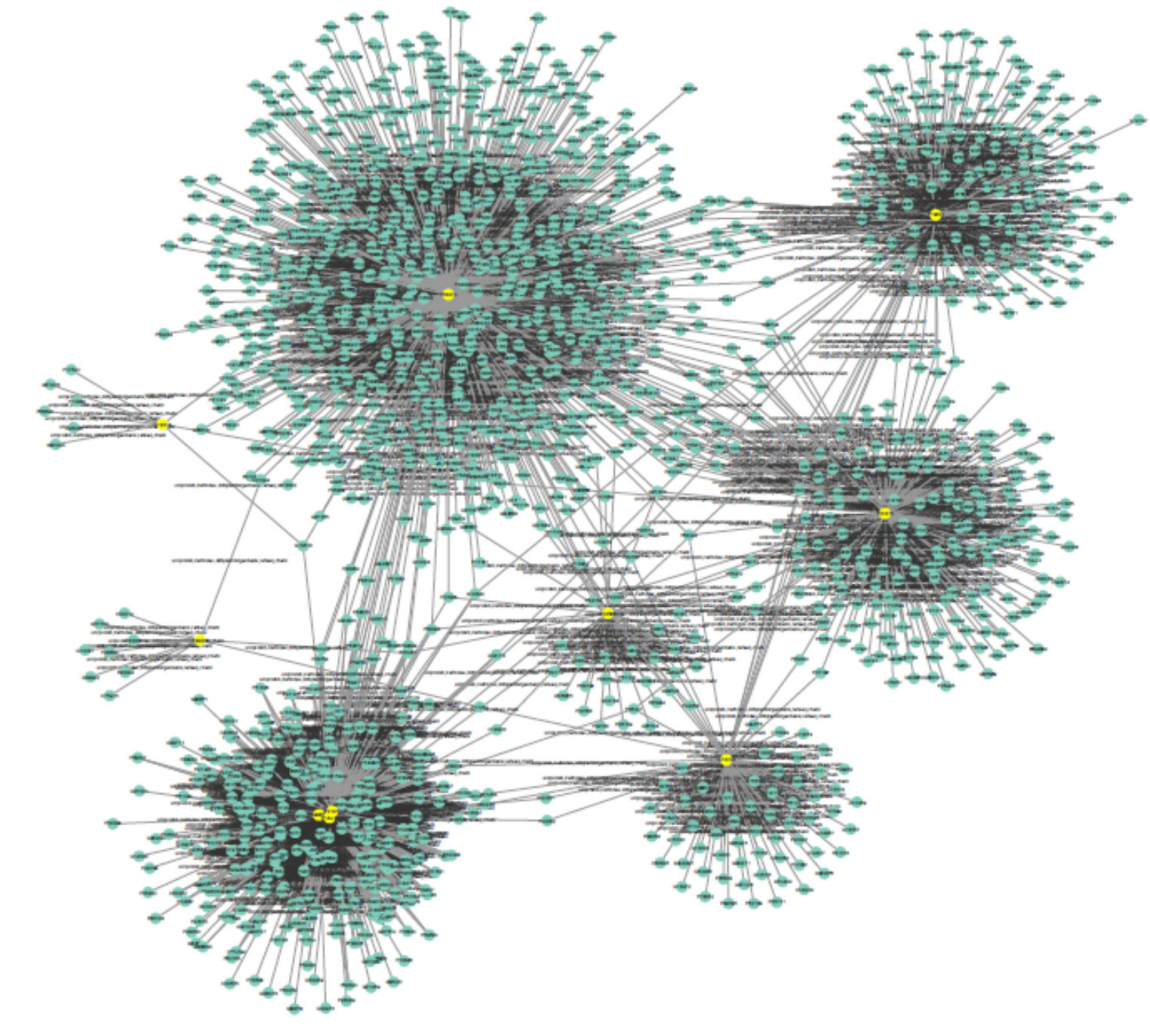
Protein-protein interaction network of ten common genes in OCD and Schizophrenia is shown. The PPI network is comprised of 1565 nodes and 2133 edges using *Cytoscape 3.3.0. *The yellow colored nodes are the ten query genes and the added genes are colored in green

**Figure 2:**
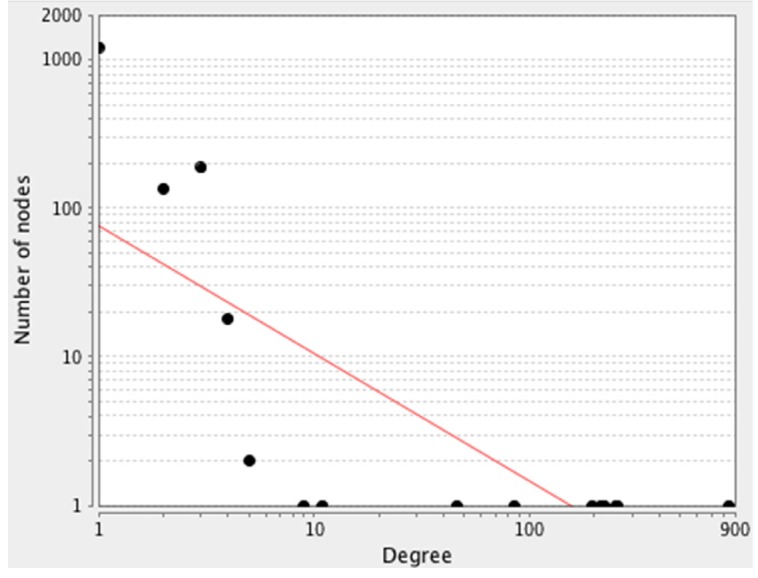
A scale-free network. The degree distribution values are significantly inhomogeneous. Just a few nodes show well linked, whereas others possess a small numbers of connections. This distribution implies on the presents of genes with high centrality values computed by Network Analyzer. The red line indicates the power law. The R-squared value is computed on logarithmized values, which is equal to 0.6 and the correlation = 0.9. Genes with high degree are in the right down region of the plot (their location is out of linear range)

Public databases including Mentha, Reactome-Fls, and Reactome were sources for PPI network construction of ten genes by the use of PSICQUIC (Proteomics Standard Initiative Common Query InterfaCe) algorithm in Cytoscape v: 3.3.0. (See Figure 1). As it is depicted in Figure 1 1555 genes are added to the 10 query genes. It is possible that the genes be screened to find the crucial individuals. The degree distribution of nodes which indicates the scale free network is illustrated in Figure 2.

The centralities evaluation provides noteworthy properties of the network topology. The genes with high value are essential elements for integrity of the network. About 20% of nodes with high degree are valuable elements and indicate hubs; similarly, 20% genes with high BC distribution imply on bottleneck genes. On the other hand, in interaction network, degree is a much better predictor of essentiality (29) (see Table 2).

**Figure 3:**
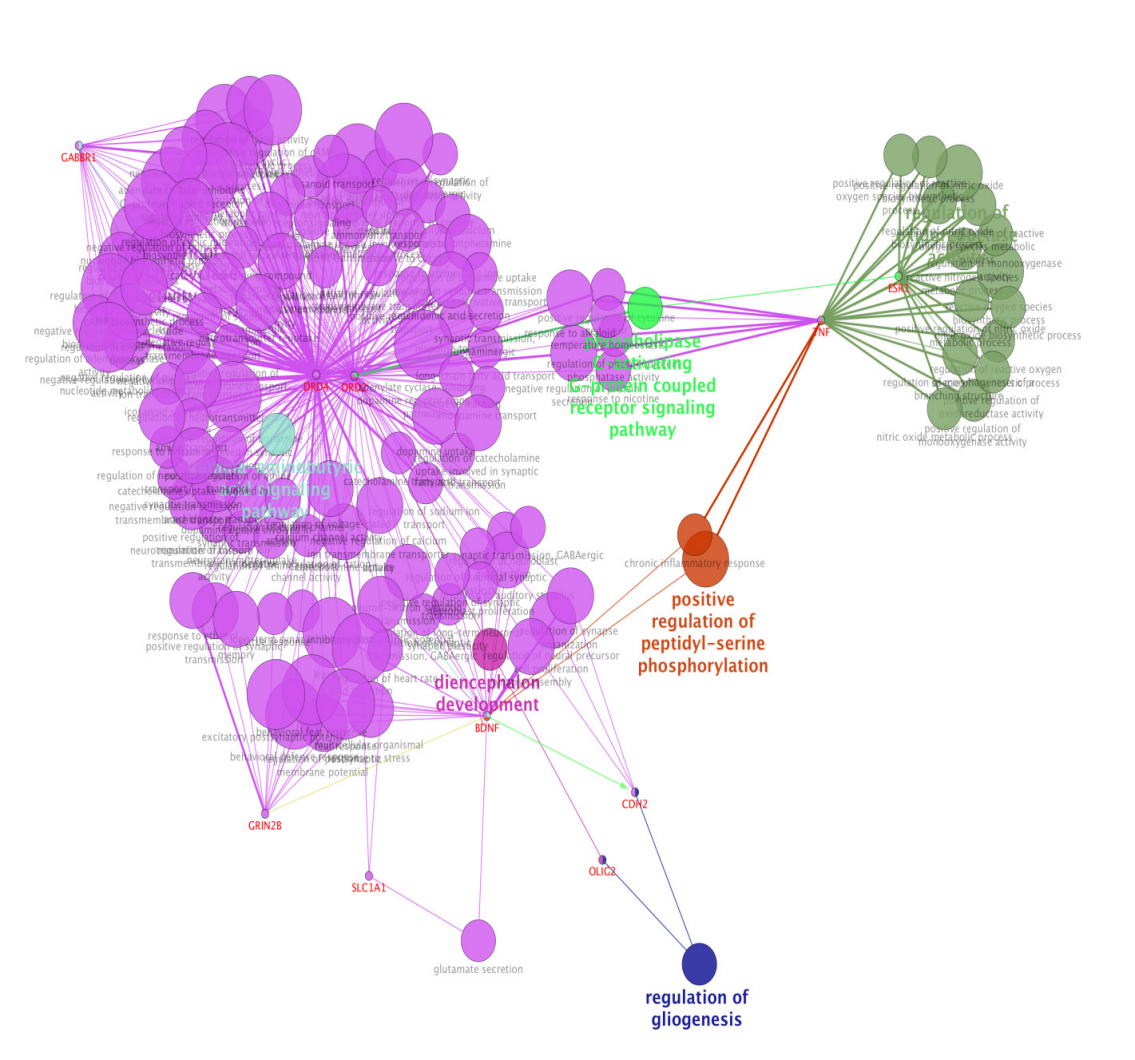
Biological process **(**BP) analysis of ten genes including their inhibition (red), activation (green), expression (yellow) characteristics by ClueGo-Cluepedia Plug-ins. The algorithm considers all the ten genes. Kappa score ≥0.5, *P*-value ≤ 0.05, gene per term = 2, gene percentage for each term = 2%

**Figure 4:**
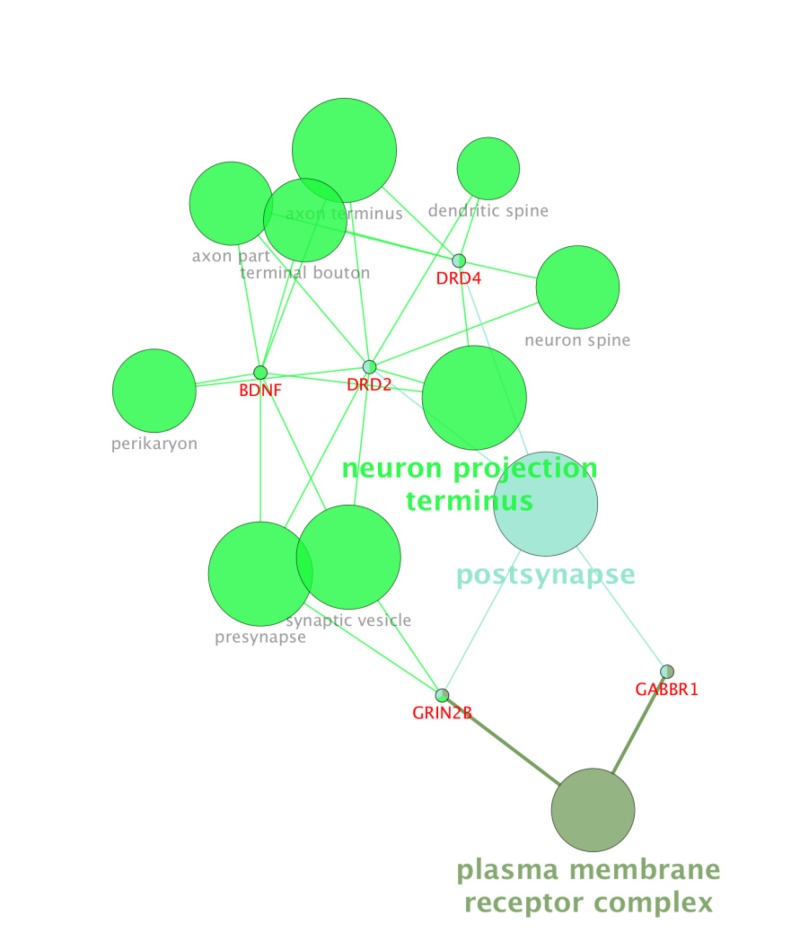
Cellular component (4) analysis of the ten genes by ClueGo-Cluepedia Plug-ins. The evaluated algorithm considered only five out of ten genes. Kappa score ≥ 0.5, *P*-value ≤ 0.05, gene per term = 2, gene percentage for each term =1%.

**Figure 5:**
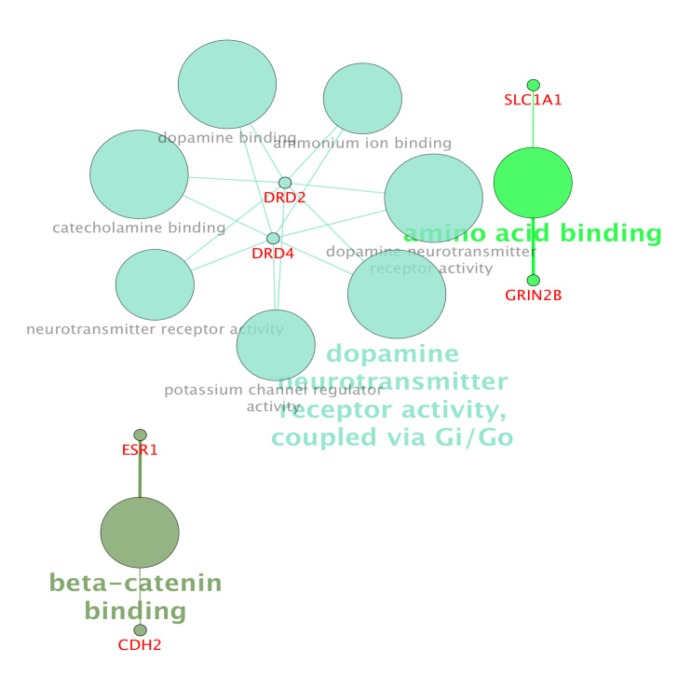
Molecular function (MF) analysis of ten investigated genes by ClueGo-Cluepedia Plug-ins. However, the evaluated algorithm considered only six out of ten genes. Kappa score≥ 0.5, *P*-value ≤0.05 , gene per term = 2, gene percentage for each term =1%.

**Figure 6:**
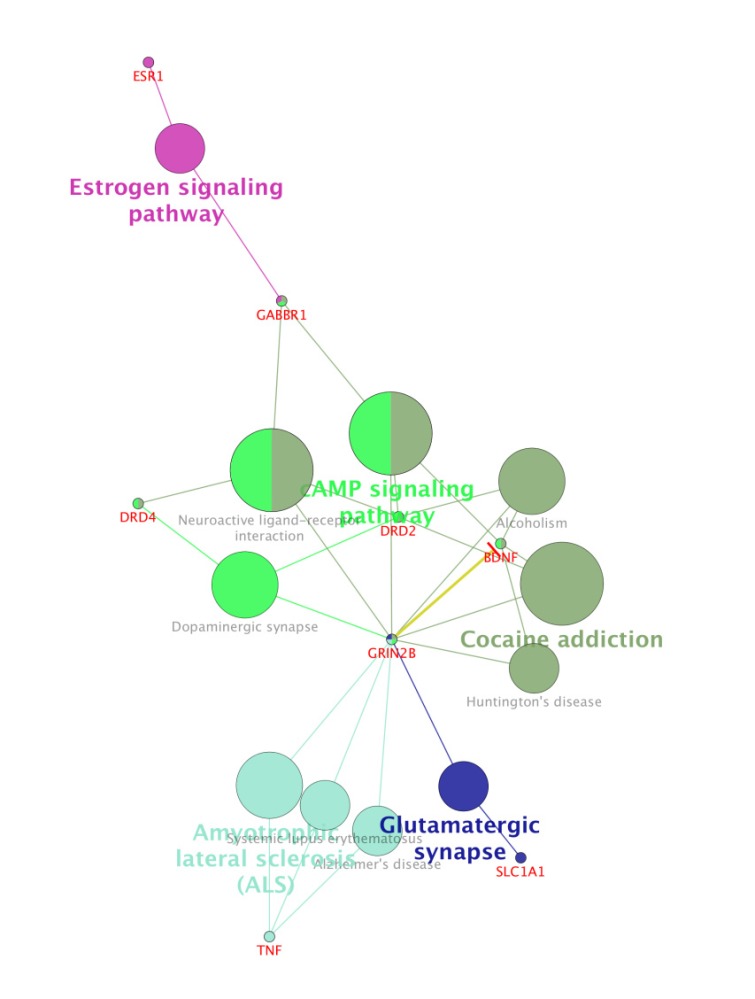
Pathway analysis of ten candidate genes by ClueGo-Cluepedia Plug-ins. KEGG Pathway and WIKIPathways were considered for pathway analysis. The evaluated algorithm considered only eight out of the ten genes. Kappa score≥0.5, *P*-value0.05 ≥ , gene per term = 2, gene percentage for each term =1%.

For more resolution, cellular component, molecular function, biological process (BP), and pathway examination of ten genes were analyzed by ClueGo-Cluepedia Plug-ins (see Figures 3-6). 

**Figure 7:**
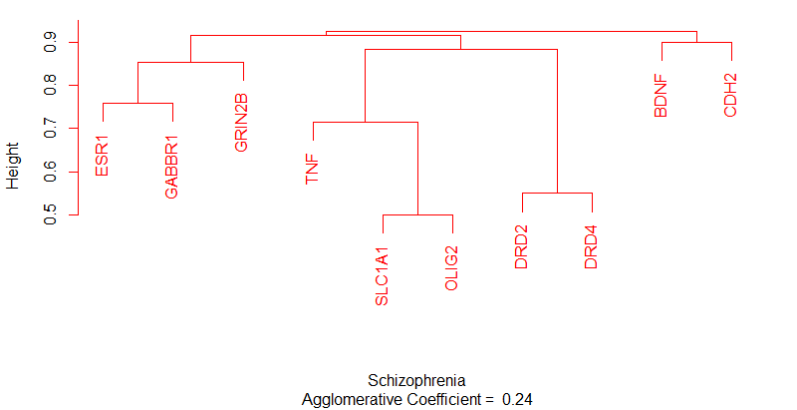
Dendrogram plotting of ten analyzed genes by the use of AGNES function. The height indicates the distance between the nodes. The agglomerative coefficient is 0.24

Estrogen receptor α (as a hub-bottleneck protein characterized with the most values of the two centrality indices that are tabulated in Table 2) is in close relationship with progesterone receptor (PGR) in the studied network. For better understanding the correlation is examined by CluePedia application of Cytoscape. The findings indicate the binding relationship between ESR1 and PGR and also activation of PGR by ESR1. Non-partitioning and partitioning clustering algorithms for sequence alignment require dissimilarity matrix. The dissimilarity matrix is calculated as (1-similarity) in which the scores are between 0 and 1. The lower the scores the shorter the distance would be between elements. The similarity and dissimilarity of the matrices of ten common candidate genes obtained from pair-wise sequence global alignment using Needleman-Wunsch algorithm from EBI databases and related dendrogram are shown in and Figure 7.

**Figure 8:**
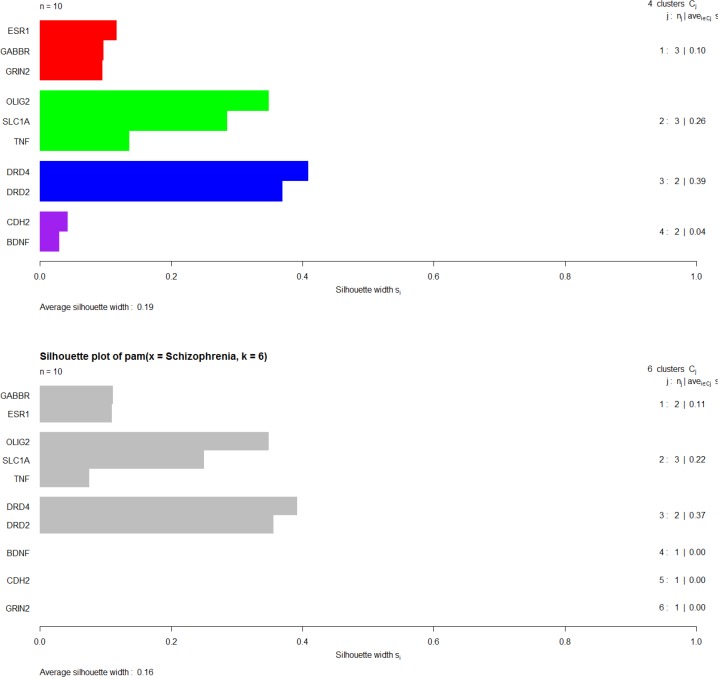
PAM clustering algorithms of ten genes via silhouette plotting, K = 4, Average silhouette width for each cluster is shown in the right side of the right column in the Figure. The overall average silhouette width is equal to 0.19

PAM clustering algorithm is done by the use of silhouette depiction. In each distinct cluster, the genes with the smallest dissimilarity average value are grouped (see Figure 8). 

**Table 1:** List of 10 common genes in OCD and Schizophrenia with their Uniprot accession codes (AC) and related protein names is presented

**R**	**Gene Names**	**Protein Names**
1	SLC1A1	Excitatory amino acid transporter 3
2	DRD2	D(2) dopamine receptor
3	DRD4	D(4) dopamine receptor
4	BDNF	Brain-derived neurotrophic factor
5	ESR1(37, 38)	Estrogen receptor
6	CDH2	Cadherin-2
7	GRIN2B	Glutamate receptor ionotropic, NMDA 2B
8	TNFa	Tumor necrosis factor
9	GABBR1	Gamma-aminobutyric acid type B receptor subunit 1
10	OLIG2	Oligodendrocyte transcription factor 2

**Table 2:** Hub-bottleneck nodes with high centrality properties (degree and betweenness) are the determinant of the essentiality of genes in the interaction network. The nodes are descended based on degree value

**Hub-bottleneck nodes**	**Degree**	**Betweenness Centrality**
ESR1	845	0.7
DRD2	257	0.1
GABBR1	224	0.1
TNFα	216	0.2
CDH2	196	0.2

**Figure 9:**
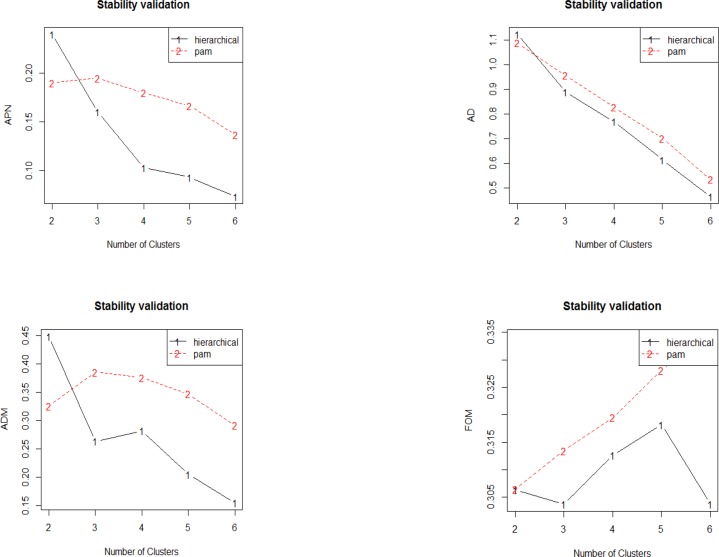
Plot of the APN, AD, ADM and FOM measures with number of cluster. The APN and AD, measures show homogenous decreased trend as the number of clusters increases for hierarchical clustering in which with six clusters has the best overall scores. For ADM, the trend decreases from two to three clusters, then there is increment to four clusters, and finally the score decreases as the number of clusters increases. In FOM, the value decreases from two to three clusters but increases afterwards to five clusters and subsequently decreased again to six clusters. Overall, hierarchical clustering has the lowest value of all evaluated indices

Dunn and Silhouette indices and also stability measure are used for assessment of validity of clustering method. Validation of clustering method via Dunn and Silhouette indices revealed optimal scores for indices with the number of clusters. Hierarchical clustering with four (score = 0.19) and six (score = 0.97) clusters performs the best in each case of Silhouette and Dunn, respectively.

The stability measures include: The average proportion of non-overlap (APN), the average distance (AD), the average distance between means (ADM), and the Figure of merit (FOM). The values should be minimized for each indices (39). Validation of clustering method via stability measure by considering APN, AD, ADM, and FOM is down. Optimal scores are provided for indices with the number of clusters. Hierarchical clustering provides the best scores for all the evaluating indices (APN, AD, and ADM) with six clusters (scores 0.07, 0.46, and 0.15 respectively) except for FOM index, which is best by three cluster (score = 0.30). 

In Figure 9, the graphically visualization provides in detail behavior of each indices by the given cluster numbers. 

Parallel and multistep analysis in this research showed that SLC1A1, DRD2, DRD4, BDNF, ESR1, CDH2, GRIN2B, TNFa, GABBR1, and OLIG2 are common genes between schizophrenia and OCD which ESR1, DRD2, GABBR1, TNFa, and CDH2 are the most important individuals. It was confirmed that ESR1 acts as a super critical genes in this regard.

## Discussion

Epidemiological studies indicated that OCD has frequent incident in schizophrenia (40). Many factors may correlate with this comorbidity. Still, the relationship between disorders remains elusive and it requires more investigations. In this light, it is important to examine the underlying etiological mechanisms to better understanding of the common molecular bases. Earlier investigations highlighted the role of common pathways of (41). Here, by exploring common significant genes between two disorders from literature, it is tried to provide a new insight of other etiological molecular concepts of the disorders. Moreover, by evaluating molecular origins including sequential and functional properties, it may be possible to achieve a better treatment protocol for the comorbid disorders. As mentioned earlier, patients with this comorbidity are more impaired in their daily life and show treatment resistance features (13). Therefore, the choice of treatment is imperative to solve this dilemma. In this regard, ten genes that showed significant association with OCD and schizophrenia were gathered from literature. At first, about thirty-nine genes for OCD were found. Among them, only twenty-four genes demonstrated significant comorbidity based on highlighting by conducted studies. For schizophrenia, there are many reported genes in schizophrenia database (42). Yet, only common OCD twenty four significant genes were evaluated. At last, ten genes (see Table 1) concluded for this study, as they were significant in schizophrenia as well. As depicted in Figure 1, Cytoscape and its plug-ins, analyzed the common significant genes and a network with 1565 nodes and 2133 edges was constructed. The PPI network shows good integrity and all the genes are in connection with each other. As showed in Table 2, centrality analysis of the network indicates that ESR1, DRD2, GABBR1, TNFα, and CDH2 possess high values in degree and betweenness centralities. In other words, these elements are Hub-bottleneck nodes are those with high value in degree and betweenness centralities. As mentioned earlier, those with high amount of degree are assigned as hubs and those with high betweenness centrality values known as bottlenecks of the network (43). These genes are critical points in the network integrity, especially, ESR1 with highest score. It is also important gene in contamination/cleaning subtype of OCD in women (44). ESR1 in schizophrenia has lower expressions in both related wild type and variants. ESR1 is involved in cognative, emotional behavior, as it is active in limbic related areas of the brain. In addition, estrogen and its receptor are vital neurosteroid for regulating many brain processes (37). One of important task of estrogen is regulating serotonin, dopamine, and norepinephrine pathways (45). ESR1 is also a DNA-binding transcription factor in cell that once bound to estrogen; it translocates to the nuclear and therefore regulates the gene expression. It is also confirmed by the high bottleneck value of ESR1 in this study that is presented in Table 2.

Notable biological processes that are pertinent to the function of ESR1and the other common genes, are shown in Figure3. The processes of regulation of oxidoreductase activity Group and phospholipase C-activating G-protein coupled receptor signaling pathway. TNFα is involved in cross-talk between positive regulation of peptidyl-serine phosphorylation, negative regulation of adenylate cyclase activity, and regulation of oxidoreductase activity. These findings are in correlation with bottleneck analysis. The bottleneck score for ESR1 and TNFα was the highest equal to 0.7 and 0.2, respectively. CDH2 is also good linker between negative regulation of adenylate cyclase activity and regulation of gliogenesis Group. BDNF gene with 0.04 betweeness score and degree of 46 is a nonhub-bottleneck node with high interconnecting of central processes including gamma-aminobutyric acid signaling pathway, negative regulation of adenylate cyclase activity and positive regulation of peptidyl-serine phosphorylation. However, the rest of important biological processes facilitated with these genes, in particular, ESR1 with highest bottleneck score are not mentioned. This fact is due to not including other network related genes in the (BP) analysis. What′s more, the BP analysis suggests that the most significant biological process in this relationship is diencephalon development. As it is shown in Figure 4, 5, and 6, neuron projection terminus, dopamine neurotransmitter receptor activity, coupled via Gi/Go and Cocaine addiction are important CC, MF and pathway in the pathogenesis of the comorbidity, respectively. As it is found in results, ESR1 has a fundamental role in activation of progesterone receptor (PGR). The genes are also in close interaction in the network. Polymorphism in ESR1 may lead to malfunction of PGR. On the other hand, studies showed that menstrual cycle is one of the important phases that OCD may trigger (46). ESR1 representation in our research may suggest the decrease of progesterone and its receptor function in this phase; it may correlate to OCD manifestation. Further analysis was the clustering analysis of sequence of the studied genes. In the clustering, it is appear that the use of AGNES and PAM was to compare their action with each other. As it is depicted in Figures 7 and 8, the cluster analysis led to similar results. However, AGNES demonstrated a more powerful cluster as calculated by cluster validation methods. ESR1 and GABBR1 are clustered together and belong to the same pathway named Estrogen signaling pathway. In addition, DRD2 and DRD4 have similarity in their sequences and they tend to contribute to similar CC, BP, MF, and pathway. However, TNF, SLC1A1, and OLIG2 genes included in a distinct cluster did not conclude to a similar corresponding biological processes. In fact, their similarity in their sequence is not very significant as the agglomerative and average silhouette width demonstrates. As mentioned earlier, general prescription for OCD patients with schizophrenia is the application of (SSRIs). Monotherapy in patients with comorbid OCD- schizophrenia may not be adequate. In fact, there are some cases with resistance responses to this type of treatment. This fact implies of the requirement of other drug affecting on central elements of the co-morbid disorders. Here, was shown that some of the key genes in the network can play fundamental role since their impairment can impose vast dysfunction in the interaction network. Additionally, among the significant associated genes, some shows higher critical role in the reactome system. ESR1 fundamental role in the network integrity suggests many clinical researches. Here, it can be concluded that in women with OCD onset in menopausal phase, the hormone therapy (estrone) or selective estrogen receptor modulators (SERM) with a tissue-specific estrogen receptor agonist/antagonist activity, may reduce the OCD symptoms. Likewise, hormone therapy and SERM proved to be effective for schizophrenia patients for both women and men (47). Indeed, estrogen deficiency and ESR1 dysfunction can increase OCD symptoms, and in menopausal state, the amount of estrogen decreases noticeably. Furthermore, many symptoms of menopause alleviates after the mentioned treatments. Yet, hormone therapy can have many side effects such as increasing the risk of breast and ovarian cancers (48, 49). Therefore, (SERMs) can be a better alternative. Based on this evidence, sources of phytoestrogens such as soy beans and flax seeds also showed promising in treatment of menopause symptoms (50, 51). Therefore, they may be useful for reducing OCD symptoms. However, it requires fundamental clinical evaluations. In addition, progesterone receptor dysfunction in patients with ESR1 polymorphism may contribute to OCD onset in the stage of menstrual activity. Therefore, the essential function of ESR1 brings out another relationship between schizophrenia and OCD in females. In other words, gender may play indispensable role in the co-occurrence of schizophrenia and OCD. Our findings were highlighted new molecular aspects of OCD-schizophrenia comorbidity. 

## Conclusion

In conclusion, regulation of ESR1 may be critical point in schizophrenia patients with OCD incident. It is suggested that the drugs which target ESR1 can be considered beside SSRIs in treatment of schizophrenia-OCD comorbidity or both disorders individually.
